# Instability of chromosome number and DNA methylation variation induced by hybridization and amphidiploid formation between *Raphanus sativus *L. and *Brassica alboglabra *Bailey

**DOI:** 10.1186/1471-2229-10-207

**Published:** 2010-09-17

**Authors:** Xuanli Li, Weiwei Guo, Bing Wang, Xiangsong Li, Honggao Chen, Lihua Wei, Yanjie Wang, Jiangsheng Wu, Hong Long

**Affiliations:** 1 College of Life Sciences and Technology, Huazhong Agricultural University, Wuhan, 430070, China; 2 National Key Laboratory of Crop Genetic Improvement, Huazhong Agricultural University, Wuhan, 430070, China

## Abstract

**Background:**

Distant hybridization can result genome duplication and allopolyploid formation which may play a significant role in the origin and evolution of many plant species. It is unclear how the two or more divergent genomes coordinate in one nucleus with a single parental cytoplasm within allopolyploids. We used cytological and molecular methods to investigate the genetic and epigenetic instabilities associated with the process of distant hybridization and allopolyploid formation, measuring changes in chromosome number and DNA methylation across multiple generations.

**Results:**

F_1 _plants from intergeneric hybridization between *Raphanus sativus *L. (2n = 18, RR) and *Brassica alboglabra *Bailey (2n = 18, CC) were obtained by hand crosses and subsequent embryo rescue. Random amplification of polymorphic DNA (RAPD) markers were used to identify the F_1 _hybrid plants. The RAPD data indicated that the hybrids produced specific bands similar to those of parents and new bands that were not present in either parent. Chromosome number variation of somatic cells from allotetraploids in the F_4 _to F_10 _generations showed that intensive genetic changes occurred in the early generations of distant hybridization, leading to the formation of mixopolyploids with different chromosome numbers. DNA methylation variation was revealed using MSAP (methylation-sensitive amplification polymorphism), which showed that cytosine methylation patterns changed markedly in the process of hybridization and amphidiploid formation. Differences in cytosine methylation levels demonstrated an epigenetic instability of the allopolyploid of *Raphanobrassica *between the genetically stable and unstable generations.

**Conclusions:**

Our results showed that chromosome instability occurred in the early generations of allopolyploidy and then the plants were reverted to largely euploidy in later generations. During this process, DNA methylation changed markedly. These results suggest that, epigenetic mechanisms play an important role in intergeneric distant hybridization, probably by maintaining a genetic balance through the modification of existing genetic materials.

## Background

Hybridization between distant genera is a driver of genome evolution and new species formation. Distant hybridization generates novel variation by causing genetic recombination [1]. Allopolyploids resulting from genome doubling during hybridization events are widespread, and include 50%-70% of angiosperms (including crops such as wheat, rapeseed, tobacco and cotton) [2]. Even though allopolyploidy is recognized as important to plant evolution [3], the processes by which it occurs are not fully understood. Coevolution and successful coexistence of genomes from diverse sources within one nuclear and only one parentaly cytoplasm requires coordinated regulation and successful genomic evolution [4-6]. Evidence from rapeseed [7,8], wheat [9-13], and *Arabidopsis *[14,15] demonstrated that allopolyploids can have rapid and extensive genomic variations, phenotypic changes, and genetic instabilities. All these genetic and epigenetic changes were inheritable; however, they did not obey the Mendelian laws of heredity [16].

Mixoploids are chimeras with different ploidies or different numbers of chromosomes existing in the same plant tissue, and include euploidy (chromosome number of offspring is the summation of two parents), hyperploidy (chromosome number of offspring is more than the summation of two parents) and hypoploidy (chromosome number of offspring is less than the summation of two parents) [17]. Hybrids of *Brassica orychophragmus *exhibit mixoploid traits [18,19].

Epigenetic changes are heritable changes in phenotype or gene expression caused by mechanisms other than changes in the underlying DNA sequence that can be perpetuated for multiple generations [20,21]. Examples of epigenetic changes include gene silencing, DNA methylation, nucleolar dominance, dormant transposon activation, and genome imprinting [22]. DNA methylation changes are detected from many neo-allopolyploids, indicating the possible role of epigenetic mechanisms in DNA methylation. Cytosine methylation changes can be detected using methylation-sensitive amplification polymorphism (MSAP), an AFLP method that uses a pair of isoschizomers, *Msp*I and *Hpa*II [23] that recognize the same restriction site (CCGG) but have different sensitivity to certain methylation states of cytosines. *Hpa*II will not cut if either of the cytosines is fully (double-strand) methylated, whereas, *Msp*I will not cut if the external cytosine is fully- or hemi-(single-strand) methylated [24]. Thus, for a given DNA sample, the full methylation of the internal cytosine, or hemi-methylation of the external cytosine at the assayed CCGG sites, can be unequivocally distinguished [23,25] using these two restriction enzymes. However, it should be noted that the methylation percentages calculated by MSAP are lower than the total absolute values at the CCGG sites [23,25,26], since *Hpa*II and *Msp*I cannot distinguish several other states of the CCGG sites, including unmethylated CCGG, fully methylated on both sites of cytosine (mCmCGG), or hemi-methylated on the internal site of cytosine (CmCGG). Nevertheless, the MSAP technique is reliable and efficient [27,28].

A distant hybrid amphidiploid, *Raphanobrassica *(2n = 2x = 36, RRCC), was synthesized by crossing two diploid species, *Raphanus sativus *L. (2n = 2x = 18, RR) and *Brassica alboglabra *Bailey (2n = 2x = 18, CC). Meanwhile, characterization of its fertility and crossability with *Raphanus sativus *and five *Brassica *species were investigated [29]. In the present paper, we used RAPD analyses to identify the genetic basis of the hybrid recovery by embryo rescue and investigated the genetic instability and epigenetic changes in this allopolyploid. Using the standard cytological chromosome squash method using ovary somatic cells, we surveyed the chromosome numbers from F_4 _to F_10 _of the allopolyploid. We also examined changes in DNA methylation of F_1 _hybrids, and the parents as well as F_4 _and F_10 _hybrids on the genome scale using MSAP. We discuss the relevance of these results to the relationship between genetic stability and epigenetic changes.

## Results

### F_1 _hybrid obtained through embryo rescue showed intermediate phenotype of the two parents

**Figure 1:**
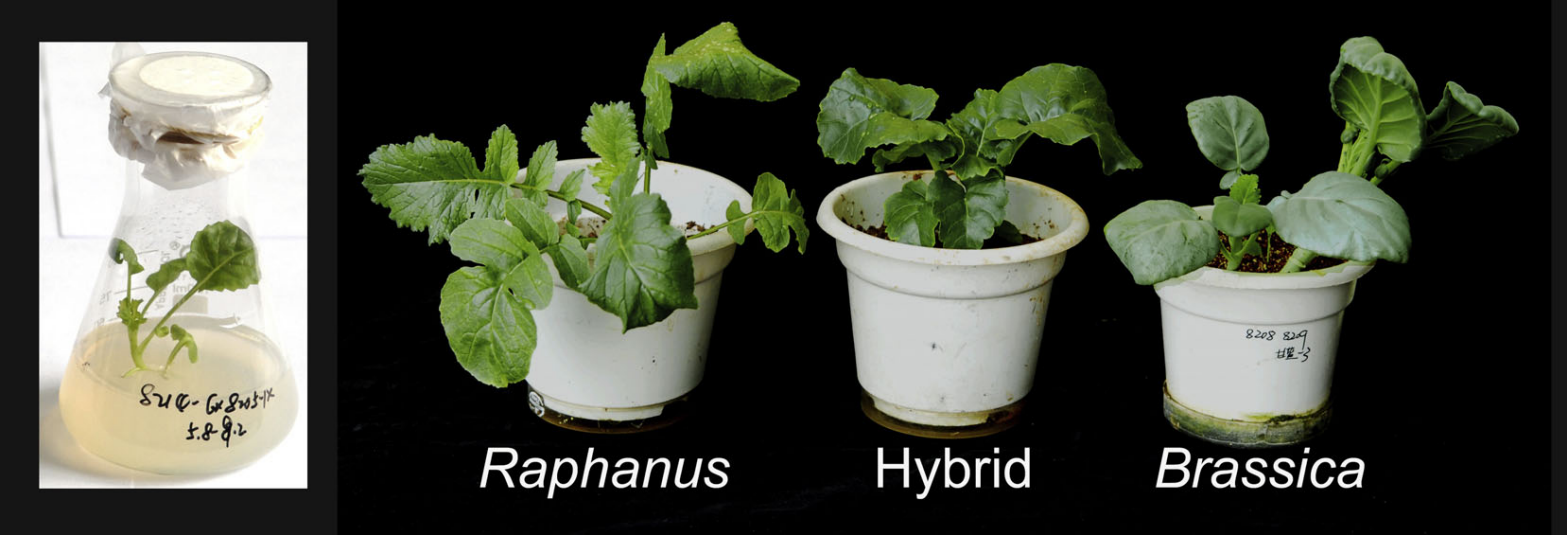
**Parents and F_1 _plants after embryo rescue**. The Erlenmeyer flask contains an F_1 _seedling 15 d after embryo rescue. Plants show the *Raphanus *and *Brassica *parents as well as an F_1 _plant 45 d after embryo rescue or sowing.

The cross between *R. sativus *cv. HQ-04 and *B. alboglabra *was made with HQ-04 as the female parent. Subsequent denudation and embryo rescue, eight F_1 _hybrids were obtained (Figure 1). The leaf morphology of the F_1 _hybrids was obviously different from that of the two parental species, indicating that these F_1 _plants are hybrids. The leaf margin of the F_1 _hybrid was lobed, with less delamination. However, leaves of female parents (*Raphanus*) and male parents (*Brassica*) were divided and indivisus, respectively (Figure 1).

### Identification of F_1 _plants with RAPD certified the genetic basis of the hybrids

**Figure 2:**
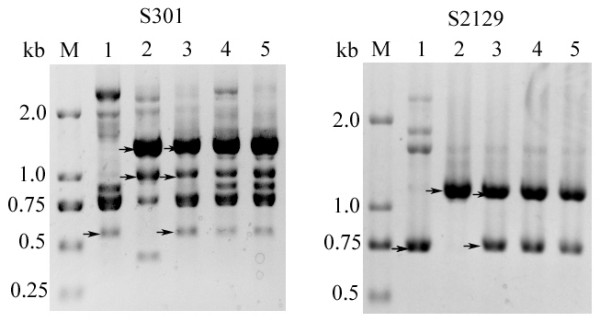
**Results of RAPD using primers S301 and S2129**. DNA marker (M) and RAPD results from *R. sativus *L. genomic DNA (lane 1 with arrow showing the specific bands of *R. sativus *L.); *B. alboglabra *Bailey genomic DNA (lane 2 with arrow showing the specific bands of *B. alboglabra *Bailey);F_1 _genomic DNA (replicated in lanes 3, 4, and 5 with arrow showing the specific bands of the parents in lane 3).

Hybrids were identified using eleven 10 bp RAPD primers (sequences provided in Additional file 1) and a total of 58 bands were amplified. Six (10.3%) of the identified bands were common to both parents and 23 bands (39.7%) were only detected in the female parent (*Raphanus*). Twenty-seven bands (46.6%) were only detected in the male parent (*Brassica*). Two bands (3.44%) were found specific to the F_1 _hybrid. These results demonstrated that the F_1 _hybrids resulted from a hybridization between *R. sativus *and *B. alboglabra*. Figure 2 shows two examples of the RAPD amplification results with primers S301 and S2129, respectively.

### Chromosome number variation of somatic cells from genetically stable F_4 _to unstable F_10 _revealed the mixopolyploidy characteristic of *Raphanobrassica *in early generations

**Figure 3:**
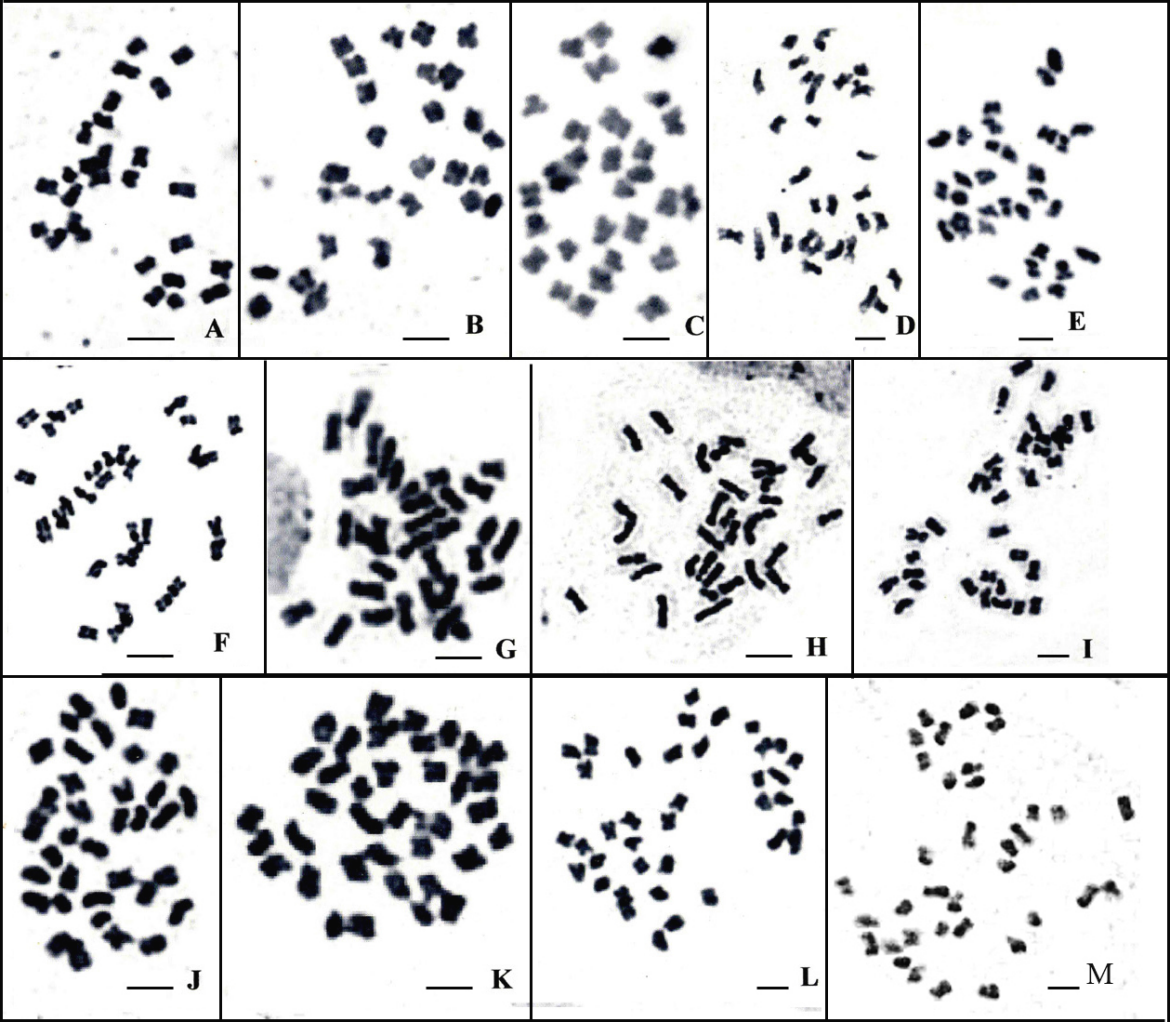
**Chromosome numbers of F_4 _and F_10 _showing their mixopolyploid and euploid character, respectively**. Chromosome numbers of F_4 _(A-L, 2n = 27-38, respectively) and F_10 _(M, 2n = 36). Bar = 5 μm.

Cytogenic cell spreads were used to compare chromosome numbers in somatic cells from ovaries to investigate the genetic instability of the artificial polyploidy. The chromosome spreading experiments were performed on six tetraploid plants in the F_4 _through F_10 _generations (Table 1). The F_4 _*Raphanobrassica *plants were identified as mixopolyploid, with chromosome numbers varying from 27 to 38 (Figure 3A-L). In all cells investigated, 50.5% were euploid (2n = 36).

**Table 1:** Somatic cell types with different chromosome numbers and the percentage in the F_4 _to F_10 _populations

		Somatic cell types													
		
Gen.	Plant No.	**38**^**a)**^	37	36	35	34	33	32	31	30	29	28	27	<27	total
F_4_	6	26^b)^	10	113	19	37	2	8	2	1	1	3	2	0	224
		11.6^c)^	4.5	50.5	8.5	16.5	0.9	3.6	0.9	0.4	0.4	1.3	0.9	0	100
F_5_	27	229	151	300	95	128	32	67	7	18	7	20	7	48	1109
		20.7	13.6	27.1	8.6	11.5	2.9	6.0	0.6	1.6	0.6	1.8	0.6	4.3	100
F_6_	27	46	120	365	235	82	9	5	7	3	0	0	0	0	872
		5.3	13.8	41.9	27	9.4	1.0	0.6	0.8	0.4	0	0	0	0	100
F_7_	32	100	105	1571	124	102	27	52	0	0	0	0	0	0	2081
		4.8	5	75.5	6	4.9	1.3	2.5	0	0	0	0	0	0	100
F_8_	30	67	111	1846	96	148	0	6	0	0	0	0	0	0	2274
		2.9	4.9	81.2	4.2	6.5	0	0.3	0	0	0	0	0	0	100
F_10_	22	0	0	119	3	0	0	0	0	0	0	0	0	0	122
		0	0	97.5	2.5	0	0	0	0	0	0	0	0	0	100

Cytogenetic cell spreading results of somatic cells from ovaries with different chromosome numbers. a) somatic chromosome number; b) cell number; c) frequency (%).

In the F_5 _generation, chromosome numbers ranged from 20 to 38, with most cells having 36 or 38 chromosomes (27.1% and 20.7% of total cells respectively), with 37, 34, 35, and 32 chromosomes being the next most frequent (Table 1). Chromosome numbers in the F_6 _generation varied from 30 to 38 and the frequency of cells with 38 chromosomes was 5.3%. The somatic chromosome numbers in F_7 _and F_8 _varied from 32 to 38, and 75.5% and 81.2% of the cells were euploid (2n = 36), respectively.

In the F_10 _generation, 119 (97.5%) cells were euploid with 36 chromosomes (Figure 3M); the three exceptional cells had 35 chromosomes.

### Extensive alteration in DNA cytosine methylation pattern was associated with formation of intergeneric hybrids

**Figure 4:**
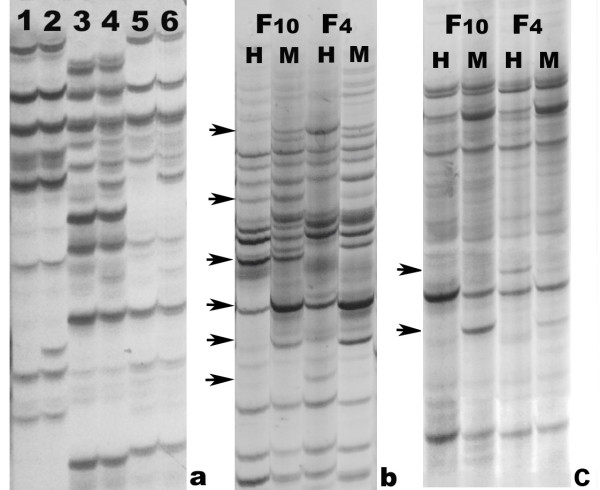
**Examples of MSAP profiles showing various types of locus-specific DNA methylation inheritance and variation in F_1 _hybrid and their parents (a) and different types of locus-specific DNA methylation inheritance and variation in F_4 _and F_10_, and the F_4 _and F_10 _DNA methylation patterns (b,c)**. (a). Primer combination is *Eco*RI+ ATT/*Hpa*II (*Msp*I) + ACC. Amplification results of *Hpa*II-digested genomic DNA of *R. sativus *L. (Lane 1), *B. alboglabra *Bailey (Lane 3) and F_1 _hybrids (Lane 5) Amplification results of *Msp*I-digested genomic DNA of *R. sativus *L. (Lane 2), *B. alboglabra *Bailey (Lane 4), and F_1 _hybrids (Lane 6). (b) and (c). Primer combinations are *Eco*RI + AGG/*Hpa*II (*Msp*I) + GAT and *Eco*RI + AGG/*Hpa*II (*Msp*I) + TCG, respectively. Arrows show variations of the various methylation patterns. H: *Hpa*II, M: *Msp*I.

In this study, MSAP was used to detect methylation changes in a site-specific manner. Eight *Eco*RI and eight *Hpa*II/*Msp*I primer pairs were tested (64 in total, see Additional file 2) and twelve primer combinations were selected, based on clear banding patterns and complete reproducibility between two independent DNA extractions from a single donor plant. The 12 pairs of *Eco*RI + *Hpa*II/*Msp*I selective primer combinations (Additional file 3) amplified by MSAP resulted in 281 clear and reproducible bands for the two parents and F_1 _hybrids (Figure 4a, Table 2).

**Table 2:** Methylation pattern in F_1 _hybrid and parents

type	male		female		**F**_**1**_		band	percentage
	*Hpa*II	*Msp*I	*Hpa*II	*Msp*I	*Hpa*II	*Msp*I		
A	0	1	0	1	0	1	5	
	1	0	1	0	1	0	2	2.49%
B	1	1	0	1	0	1	17	
	0	1	1	1	0	1	2	
	1	1	0	0	0	0	27	
	0	0	1	0	0	0	4	
	0	0	0	1	0	0	3	
	0	0	1	0	1	0	4	
	0	0	1	1	0	0	25	
	1	1	0	0	1	1	54	
	1	1	0	1	1	1	9	
	0	0	0	1	0	1	10	
	0	0	1	1	1	1	62	
	1	0	1	0	0	0	1	
	0	1	1	0	1	0	2	
	0	1	0	0	0	1	6	
	1	1	1	0	1	0	1	80.78%
C	1	0	1	0	0	0	1	
	0	1	0	1	0	0	1	
	1	1	1	1	0	0	7	
	1	1	1	1	0	1	4	
	1	1	1	1	1	0	1	
	1	1	0	1	0	0	4	
	1	0	1	1	0	0	1	
	0	1	1	1	0	0	2	7.47%
D	0	0	0	0	1	0	1	
	0	0	0	0	0	1	3	
	0	0	0	0	1	1	5	
	0	1	0	1	1	1	8	
	0	1	0	0	1	1	5	
	0	0	1	0	1	1	3	
	0	0	0	1	1	1	1	9.25%
total							281	

DNA methylation patterns in F_1 _hybrid and parents. "0" stands for the absence of a band, and "1" stands for the presence of a band.

DNA methylation sites in F_1 _hybrids result in patterns observed to be inherited from one or both parent (s) or induced by intergeneric hybridization, including hyper- or hypo-methylation. The MSAP results showed that methylation sites of the parents were transmitted to F_1 _hybrid mainly in a Mendelian way, about 83.27% (2.49% from both parents, type A; 80.78% from only one parent, type B). Meanwhile, 16.72% were variant bands (7.47% were hypermethylation, type C; 9.25% were hypomethylation, type D).

The small number of methylation sites that were similar to both the parents was probably due to the fact that the methylation states in the parents of different genera were not the same. The bands coming from only one parent, i.e. type B, comprised a much larger percentage. Type C (hypermethylation) patterns were slightly less than type D (hypomethylation), indicating that the level of F_1 _methylation may be lower than that of the parents.

### Difference in cytosine methylation levels showed epigenetic instability of allopolyploid of *Raphanobrassica *between genetically stable and unstable generations

From the cytogenetic analysis above, we concluded that the genetic instability grew from F_4 _to F_10_, with most somatic cells having 36 chromosomes. The F_4 _generation was typically unstable while the F_10 _generation was more stable. Since epigenetic mechanisms may play an important role in the correlation of nuclear-cytoplasm system in allopolyploids, we examined DNA methylation changes between F_4 _and F_10_.

With 12 pairs of *Eco*RI+ *Hpa*II/*Msp*I selective primer combinations (Additional file 3), 414 and 432 clear and reproducible bands for the two generations were amplified by MSAP (Table 3).

**Table 3:** Levels of cytosine methylation at the CCGG sites of F_4 _and F_10 _of allopolyploid of *Raphanobrassica*

			Methylated sites (number and frequency)		
			
Generations	Total sites	Non-methylated sites (number and frequency)	total	hemi-methylation of the external Cs	full-methylation of the external Cs
F_4_	432	306 (70.83%)	126 (29.17%)	87 (20.14%)	39 (9.03%)
F_10_	414	283 (68.36%)	131 (31.64%)	83 (20.05%)	48 (11.59%)

Methylated and non-methylated sites (number and frequency in the F_4 _and F_10 _generations) were calculated according to the clear and reproducible bands of MSAP.

Between the F_4 _and F_10 _generations, the total methylation levels (calculated by adding up the various patterns) increased from 29.17% to 31.64%. This includes 9.03% (F_4_) and 11.59% (F_10_) hemi-methylation of external C, 20.14% (F_4_) and 20.05% (F_10_) full-methylation of the internal C (Figure 4b, c, Table 3).

Based on the MSAP patterns, various bands representing non-methylation, hemi-methylation of external C, full methylation of internal C, and full methylation of external C or both Cs (see *Introduction *for the rationale of band scoring) were tabulated (Table 4). Fourteen types of amplified bands were obtained after comparing DNA methylation states of F_4 _and F_10 _(Table 4). These bands were divided into two groups: group I showed monomorphic sites, which had no difference in F_4 _and F_10_, while group II showed polymorphic sites, which had a difference between the two generations. There were two types of bands in group I: IA was full-methylation of the internal C (*Hpa*II do not cut/*Msp*I cut, H/M = -/+), and IB was hemi-methylation of the external C (*Hpa*II cut/*Msp*I do not cut, H/M = +/-). The total number of these bands was 102 (Table 4), indicating that 58.29% (102/175) of DNA sequences had no changes in methylation between the F_4 _and F_10 _generations. These results indicated that most methylation types could be stably transmitted from one generation to the next. Group II was divided into 12 types, suggesting that there were some DNA methylation changes between the two generations (Table 4). Some bands were only detected in F_4_, while others were only detected in F_10 _(Figure 4b, c). A total of 71 bands like these could be divided into 5 sections (Table 4, IIA, IIB, IIC, IID, IIE). Among these, IIA and IIB were the most abundant, with 27 and 18 bands, respectively. Section IIA showed no bands in both or one of *Hpa*II/*Msp*I digested lanes in F_4_, while it had bands in F_10_. These patterns (IIA-1, - - + +, IIA-2, - + + + IIA-3, + - + +) indicated full hypomethylation in F_10_, present for 15.43% of the total. In contrast, Section IIB had bands in both or one of the *Hpa*II/*Msp*I digested lanes in the F_4_, and no bands were produced in F_10_. These patterns (IIB-1, + + - -, IIB-2, + - - -, IIB-3, - + - -) indicated a full methylation of external C or both Cs in the F_10_, present in 10.29% of the total. Sections C, D, and E showed hypo- and hypermethylation states in F_10 _compared to the F_4_, that had 6.29%, 5.71%, and 4.00%, respectively.

**Table 4:** Different patterns of methylation in F_4 _and F_10_

Patterns			**F**_**4**_		**F**_**10**_		Number of sites	Ratio of different patterns (%)
					
			H	M	H	M		
I		IA	-	+	-	+	74	58.29
		IB	+	-	+	-	28	
II	A	IIA1	-	-	+	+	10	15.43
		IIA2	-	+	+	+	6	
		IIA3	+	-	+	+	11	
	B	IIB1	+	+	-	-	3	10.29
		IIB2	+	-	-	-	7	
		IIB3	-	+	-	-	8	
	C	IIC1	-	-	-	+	3	6.29
		IIC2	-	-	+	-	8	
	D	IID1	+	+	-	+	8	5.71
		IID2	+	+	+	-	2	
	E	IIE1	+	-	-	+	2	4.00
		IIE2	-	+	+	-	5	

DNA methylation number of sites and ratio of different patterns. "- " stands for the absence of a band, and "+" stands for the presence of a band. H and M are the combinations of enzymes of *Eco*RI/*Hpa*II and *Eco*RI/*Msp*I, respectively.

## Discussion

Distant hybridization occurs under both natural and artificial conditions. After genome duplication, allopolyploids can be obtained, and these polyploids play a significant role in the origin and evolution of many plant species. In crop improvement, allopolyploids are also valuable as bridge materials for breeding. During the course of hybridization, it is difficult to get F_1 _plants due to sterility. The F_1 _hybrid could be the real hybrid containing whole or partial genetic material from both of the parents; the false hybrid resulting from female parthenogenesis or self-crossing; or an introgression where most of the male parent DNA was digested by the female nuclease and a few segments combined with the genome of the female parent.

Allopolyploidy has been intensively studied in naturally evolved allopolyploids of wheat, cotton, rapeseed, *Arabidopsis*, and tomato. Genetic changes are best evaluated by comparing the parental lines and their progeny; however, it is difficult to ascertain the parents in naturally evolved allopolyploids since genetic changes have occurred in subsequent generations. Artificial allopolyploids have defined parents and genetic lineages, making them suitable for elucidating changes that occur during the formation of allopolyploids.

The hybridization between *Raphanus *and *Brassica *was first reported by Sageret in 1862 [30]. About 100 years later, Karpechenko identified an F_1 _hybrid between *Raphanus *and *Brassica *[31,32]. To transfer the nucleic genes of *Brassica tournefortii *(TT) into *B. carinata *(BBCC), Mukhopadhyay et al. produced a bridge species (TCBB) by protoplast fusion between the F_1 _hybrid (TC) of *B. tournefortii*×*B. oleracea *and *B. nigra *(BB) and RAPD primers were used to show that all the hybrids had specific bands from the genomes of the parents. These results indicated that the T, B, and C genomes may coexist in a hybrid state. RFLP molecular markers confirmed that these hybrids contained chloroplast and mitochondrion genomes of *Brassica tournefortii *and *B. nigra *[33]. Chrungu et al. (1999) synthesized allotetraploids of *B. maurorum*-*B. napus*, *B. maurorum*-*B. carinata*, and *B. maurorum*-*B. nigra *through interspecific hybridization and genomic doubling and proved the reality of the hybrids through RAPD and RFLP [34]. In the present study, we synthesized F_1 _hybrids through sexual hybridization followed by embryo rescue and identified its origin with RAPD. The results showed that F_1 _hybrids lost some of the specific or common bands of the parents. The probable reason was that hybridization resulted in a genomic recombination and changed the primer binding sites of some segments, leading to the appearance or disappearance of some DNA bands.

Our results showed that, DNA methylation patterns differed substantially between the parents and F_1 _hybrids, indicating its possible key role in transgenerational stability. During the process of hybrid formation, DNA methylation patterns may adjust to some extent to coordinate the interactions among nuclear genes or those between nuclear genes and cytoplasmic genes. Both hypomethylation and hypermethylation events may affect gene expression patterns. In our work, hypomethylation was a more frequent than hypermethylation.

Polyploidy has played a fundamental role in the evolution of higher plants. Plant breeders have been routinely producing allopolyploids with interesting agronomic traits to be used for breeding programs. However, the poor genetic stability of allopolyploids in early generations is a challenge for plant breeding programs.

Intergeneric hybridization between a long genetic distance may cause mixoploidy, leading to genetic instability. Hybrids between *Orychophragmus violaceus *(2n = 24) and cultivated *Brassica *species, including tetraploids (*B. carinata *and *B. juncea*) and diploids (*B. campestris *and *B. nigra*), led to mixoploids [18,19]. For example, the hybrid with *B. campestris *(2n = 20, AA) was mixoploid (2n = 23-42), and cells with 2n = 34 were most frequent. Partial separation of parental genomes during mitosis, leading to the addition of *O. violaceus *chromosomes to the *B. campestris *complement, was proposed to explain the findings in the mitotic and meiotic cells of the hybrid and its progeny. In crosses with *B. nigra *(2n = 16, BB), a small fraction consisted of mixoploids (2n = 16-18), predominantly with 2n = 16 cells, and three plants, each with a specific morphology, were mixoploids consisting of cells with varying ranges of chromosome numbers (2n = 17-26, 11-17 and 14-17). The origin of these different types of plants was inferred to be the result of complete and partial separation of parental genomes and the loss of *O. violaceus *chromosomes.

In the present study, our intergeneric hybrid of *R. sativus *L. and *B*. *alboglabra *Bailey was a mixoploid (2n = 23-42) in the early generations (F_4_-F_8_). After several generations, mixoploids gradually turned to euploids through formation of neo-chromosomes or chromosome elimination. The possible cytological mechanisms pertaining to these hybrid generations and the genetic motifs from unstable to stable generations are unknown

We suggest that the stability of the F_10 _generation is a result of epigenesist. Epigenetic changes were mainly materialized by covalent modifications of DNA methylation and protein modifications (methylation, acetylation, phosphorylation). In plants, newly acquired epigenetic states of transcriptional gene activity can be readily transmitted to the progeny through meiosis. Epigenetic reconfiguration after hybridization between diploid members of the same species may be an important mechanism for reconciling two non-identical genomes in the same nucleus as allopolyploid formation occurs [5,35,36].

Our results show that methylation patterns and methylation states changed in the intergeneric hybrid both in the process of F_1 _hybrid formation and through the formation of generations of genetically unstable and stable progenies. DNA methylation levels of F_4 _and F_10 _(29.17% and 31.64%) did not show a great difference, indicating the similar status of DNA methylation between stable and unstable generations. These levels were similar to that of *Arabidopsis *seedlings (35%-43%) [37], but these were much higher than that of rice leaves (16.30%) [38]. These differences in DNA methylation among different species may be caused by detection methods (primer numbers, amplification conditions, time of electrophoresis, and staining methods), material differences (seed, seedling, mature leaves), or genetic control. For DNA methylation patterns, hemi-methylation levels of the external cytosine at CCGG sites of F_4 _and F_10 _are 20.14% and 20.05%, respectively, while full methylation of the internal cytosine at CCGG sites of F_4 _and F_10 _were 9.03% and 11.59%, respectively. These results showed that hemi-methylation was the main DNA methylation pattern in both F_4 _and F_10 _generations. The full-methylation level of the F_10 _was higher than that of the F_4_, indicating that methylation changes from an unstable to a stable generation happened mainly on full-methylation sites. The relationship between the changes in genetics and epigenetics remains elusive. We suggest that the inheritance of allopolyploids seems to be governed by multiple mechanisms, including both genetic and epigenetic mechanisms. The contribution of these different mechanisms to inheritance is largely unknown, as many of these interact with each other.

## Conclusions

Wide hybridization followed by genome doubling is a prominent model of speciation in higher plants. Nevertheless, little is known regarding the early events of coordination required to ensure compatibility for the coexistence of two or more divergent genomes derived from different species in a single nucleus with one parental cytoplasm. Here we presented evidence of genetic and epigenetic variation resulting from hybrid formation. Although a deeper insight into these changes is still needed, epigenetic, especially DNA methylation patterns and levels, is undoubtedly one of the important factors in controlling allopolyploid formation and development. One can expect that the multiple, comparative analyses of biological events during the formation of hybrids will ultimately facilitate an inference of the rules and principles that control the fate of duplicated genes and will lead to an enhanced appreciation of the effects of distant hybridization on the formation and the natural evolution of new species.

## Methods

### Plant materials

Selfed generations of *Raphanus sativus *cv. HQ-04 (a vegetable radish landrace in Wuhan) and *Brassica alboglabra *were used to synthesize amphidiploid *Raphanobrassica*. The seeds were preserved at the National Rapeseed Engineering Center, Huazhong Agricultural University, Wuhan, China.

### Field hybridization

Planting was carried out in the field of the National Rapeseed Engineering Center, Huazhong Agricultural University, Wuhan, China. The cross between *R. sativus *cv. HQ-04 and *B. alboglabra *was made with HQ-04 as the female parent. For production of F_1 _hybrid, the female parents were denudated buds, castrated, and bagged. Male parents were also bagged 1 d before flowering. After flowering, pollinations were performed, followed by bagging and labelling with tags to record date and hybrid combination.

After embryo rescue (see below), F_1 _plants were treated with 0.3% aqueous colchicine to double the chromosome number. After two generations of self-pollination, six partially fertile putative amphidiploid plants (2n = 36) with intermediate morphological characters were selected from 41 F_4 _plants. Seeds harvested from these plants after self-pollination were planted and 385 F_5 _plants were generated. They were permitted to pollinate each other within a mosquito nylon net. Six F_5 _plants with higher fertility were selected. Using similar procedures, selections were done until the F_10 _generation.

### Embryo rescue

At 5, 9, and 13 d after fertilization, ovaries were cut, surface-sterilized with 75% alcohol for 0.5 min, immersed in 0.1% mercuric chloride for 12 min, and washed with distilled water three times. Inoculation was done using the medium MS+0.2 mg/L 6-BA, 25-27°C, and daylight of 16 h with 800 1ux. After 20 d, young embryos were separated and transferred to MS medium until the seedlings grew.

### Genomic DNA isolation and RAPD

Seeds of *R. sativus*, *B. alboglabra*, F_4 _and F_10 _were planted in a Sanyo growth cabinet (Osaka, Japan) after surface sterilization with 75% alcohol as described above. Genomic DNA was isolated from expanded leaves in parental, F_1_, and subsequent generations of plants using a modified CTAB method [39] and a phenol purification. Quality and quantity of DNA were inspected by both gel electrophoresis and spectrometric assays. One hundred random primers with 10 bp were selected for PCR amplification of genomic DNA from the parents and F_1 _hybrid. The thermal cycles started with 94°C for 5 min; then 40 cycles of 94°C for 1 min, 37°C for 1 min, 72°C for 1 min, ending with 72°C for 7 min. The amplification products were separated on 1.5% agarose gel with 0.5 μg/ml ethidium bromide in 0.5×TBE buffer at 100 V for 3.5-4 h and photographed under ultraviolet light.

### Cytological analysis

Ovaries collected from each plant were used for cytological study following the procedure of Li et al. [18] with modifications. Briefly, materials were dissected under a stereomicroscope and treated with 0.002 mol/L 8-hydroxyquinoline for 3 h in total darkness at room temperature, followed by fixation Carnoy's fixation solution (ethanol: acetic acid = 3: 1, v/v) for at least 1 h at room temperature. The materials were macerated in 1 mol/L hydrochloric acid at 60°C for 10 min. After washing three-five times to eliminate residual hydrochloric acid and staining with carbol fuschin for 1 min, the materials were squashed for observation in 45% acetic acid. More than 30 chromosome micrographs were observed under an Olympus BX61 microscope and recorded with spot pursuit slider digital camera (USA). A total of 224 somatic cells were observed from from ovaries of six plants in F_4 _generation and chromosomes of 1,109 somatic cells from ovaries of 27 plants in the F_5 _were investigated. Chromosomes of 872 somatic cells from ovaries of 27 plants in F_6 _and 122 cells from ovaries in 22 plants of the F_10 _were observed.

### MSAP analysis

MSAP, a technique based on AFLP, was used to detect methylation changes on specific sites [22].

A pair of isoschizomers, *Hpa*II/*Msp*I, was used instead of *Mse*I in AFLP. *Hpa*IIand *Msp*I form a pair of isoschizomers that recognize the same restriction site (5'-CCGG) but have different sensitivity to methylation of the cytosines.

The MSAP protocol used in this study was essentially as reported previously [23]. The restriction enzymes *Eco*RI, *Hpa*II, and *Msp*I were purchased from the New England Biolabs Inc. (Beverly, Mass.). In brief, one pair of pre-selective primers and 16 pairs of selective primers were used for amplifications. A silver-stained sequencing gel was used to resolve and visualize the amplification products. Only clear and completely reproducible bands that appeared in two independent PCR amplifications (starting from the digestion-ligation step, i.e., the first step of MSAP) were scored. The scored MSAP bands represent three major cytosine methylation states: (1) hemi-methylation of the external C, which are bands present in *Hpa*II but absent from the corresponding *Msp*I-digest, i.e., pattern H/M = +/-; (2) full methylation of the internal C, which are bands absent from *Hpa*II but present in the corresponding *Msp*I-digest, i.e., pattern H/M = -/+, and; (3) full methylation of the external C or both Cs, which are bands absent from both *Hpa*II- and *Msp *I-digest but present in the alternative tissue of the same genotype, i.e., pattern H/M = -/- in tissue1 versus H/M = +/+ in tissue2, and vice versa.

## Authors' contributions

XuL, XiL, LW, YW, and BW carried out the molecular genetic studies. BW and WG participated in the sequence alignment and drafted the manuscript. HC, BW, and HL carried out the cytological analysis. LW and JW participated in the design of the study. HL designed and finalized the manuscript. All authors read and approved the final manuscript.
